# Antioxidant effects of insulin-like growth factor-I (IGF-I) in rats with advanced liver cirrhosis

**DOI:** 10.1186/1471-230X-5-7

**Published:** 2005-03-03

**Authors:** María García-Fernández, Inma Castilla-Cortázar, Matías Díaz-Sanchez, Iñigo Navarro, Juan Enrique Puche, Alberto Castilla, Amelia Díaz Casares, Encarna Clavijo, Salvador González-Barón

**Affiliations:** 1 Department of Physiology, School of Medicine, University of Navarra, Pamplona, Spain; 2 Department of Human Physiology. School of Medicine, University of Málaga, Spain; 3 Department of Physiology, School of Medicine, University of San Pablo-CEU, Madrid, Spain; 4 Department of Chemistry, School of Medicine, University of Navarra, Pamplona, Spain; 5 Department of Internal Medicine, Hospital Sierrallana, Torrelavega. Cantabria, Spain; 6 Department of Microbiology, School of Medicine, University of Málaga, Spain

## Abstract

**Background:**

The exogenous administration of Insulin-like Growth Factor-I (IGF-I) induces hepatoprotective and antifibrogenic actions in experimental liver cirrhosis. To better understand the possible pathways behind the beneficial effect of IGF-I, the aim of this work was to investigate severe parameters involved in oxidative damage in hepatic tissue from cirrhotic animals treated with IGF-I (2 μg. 100 g^-1^. day^-1^). Iron and copper play an important role in oxidative mechanisms, producing the deleterious hydroxyl radical (*OH) that peroxides lipid membranes and damages DNA. Myeloperoxidase (MPO) and nitric oxide (NO) are known sources of free radicals and induce reduction of ferritin-Fe^3+ ^into free Fe^2+^, contributing to oxidative damage.

**Methods:**

Liver cirrhosis was induced by CCl_4 _inhalation in Wistar male rats for 30 weeks. Healthy controls were studied in parallel (n = 10). Fe and Cu were assessed by atomic absoption spectrometry and iron content was also evaluated by Perls' staining. MPO was measured by ELISA and transferrin and ferritin by immunoturbidimetry. iNOS expression was studied by immuno-histochemistry.

**Results:**

Liver cirrhosis was histologically proven and ascites was observed in all cirrhotic rats. Compared to controls untreated cirrhotic rats showed increased hepatic levels of iron, ferritin, transferrin (p < 0.01), copper, MPO and iNOS expression (p < 0.01). However, IGF-treatment induced a significant reduction of all these parameters (p < 0.05).

**Conclusion:**

the hepatoprotective and antifibrogenic effects of IGF-I in cirrhosis are associated with a diminution of the hepatic contents of several factors all of them involved in oxidative damage.

## Background

Insulin-like growth factor-I (IGF-I) is an anabolic hormone produced in different tissues in response to growth hormone (GH) stimulation [1]. Liver synthesis of IGF-I accounts for 90% of the circulating peptide. In cirrhosis the reduction of receptors for GH in hepatocytes and the diminished synthesis ability of the hepatic parenchyma cause a progressive fall in serum IGF-I levels. The clinical impact of the decreased in IGF-I production in advanced cirrhosis is largely unknown [2-5]. Recent studies from our laboratory in rats with carbon tetrachloride-induced cirrhosis have demonstrated that short courses of treatment with low doses of IGF-I are able to produce systemic beneficial effects [6-13] and are associated to hepatoprotective [14,15] and antifibrogenic [16] effects.

In order to give a better insight into the pathways by which IGF-I seems to exert its the hepatoprotective and antifibrogenic actions, this study was aimed at analyze several parameters involved in oxidative stress or inflammation in the liver, such as metals ions (iron and copper), iron transport and store proteins (transferrin and ferritin) and enzymes (myeloperoxidase -MPO- and inducible nitric oxide synthase -iNOS-) both in IGF-I treated and untreated cirrhotic rats.

Metal ions, such as iron and copper, exhibit the ability to produce reactive oxygen species, resulting in lipid peroxidation, DNA damage, depletion of sulfhydryls and altered calcium homeostasis [17-19]. Iron-dependent processes play a pivotal role in the development of oxidative-induced cell injury. Specifically, the generation of hydroxyl radicals from hydroperoxide and the formation of aldehydes and lipid peroxy radicals from lipid hydroperoxides are catalyzed by redox-active metals, including iron and copper [17,20,21]. MPO and NO are known sources of free radicals and induce reduction of ferritin-Fe^3+ ^into free Fe^2+ ^contributing to oxidative damage [22,23].

## Methods

### Induction of liver cirrhosis

Cirrhosis was induced as previously described [9,12]. Briefly, male Wistar rats (3 weeks old, 130–150 g) were subjected to CCl_4 _inhalation (Merck, Darmstadt, Germany) twice a week for 11 weeks with a progressively increasing exposure time from 1 to 5 minutes. From that time until the 30^th ^week rats were exposed to CCl_4 _once a week for 3 min. During the whole period of cirrhosis induction animals received Phenobarbital (Luminal, Bayer, Leverkusen, Germany) in the drinking water (400 mg/L). Rats were housed in cages placed in a room with 12-hour light-dark cycle and constant humidity and temperature (20°C). Both food (standard semipurified diet for rodents; B.K. Universal, Sant Vicent del Horts, Spain) and water were given *ad libitum*. Healthy, age and sex-matched control rats were maintained under the same conditions but receiving neither CCl_4 _nor Phenobarbital.

All procedures were performed in conformity with *The Guiding Principles for Research Involving Animals *[24].

### Study design

The treatment was administrated the last three weeks (27^th ^-30^th^) of CCl_4 _exposure (from day 0 to day 22^nd^). In the morning of day 0, animals were weight and blood samples were drawn from the retroocular venous plexus from all rats with capillary tubes (Marienfeld, Germany) and stored at -20°C until used for analytical purposes. Cirrhotic rats were randomly assigned to receive either vehicle (saline) (CI, n = 10) or recombinant human IGF-I (Pharmacia-Upshon, Sweden) (2 μg × 100 g bw ^-1 ^× day ^-1 ^in two divided doses, subcutaneous) (CI+IGF, n = 10) for three weeks. Control rats (CO, n = 10) received saline during the same period. The last dose of IGF-I was administrated the day 21^st ^at 6 p.m.

In the morning of the 22^nd ^day, animals were weight and killed by decapitation. After the abdominal cavity was opened, the liver was dissected and weight. A sample from the left major liver lobe was processed for histological examination (fixed in Bouin's solution). The rest of liver samples were stored at -80°C.

### Liver histopathology, Perls'stain and immunohistochemistry

Bouin-fixed tissues were processed and sections (4-μm.) were stained with Haematoxylin and Eosin and Masson's trichrome. Liver cirrhosis was diagnosed according to the criteria previously described [14,16]. Liver sections were stained for iron detection with Perls' Prussian Blue [25,26]. A semiquantitative score was given since 0 to 6 points: 0 when no staining was observed, as it was observed in controls; 6 points were assigned to sections with the maximal staining (full staining), that it was observed in liver macrophages and fibrous septa from cirrhotic rats; 2–5 points when the staining were less extent. Four fields from each preparation (×100 magnification) were evaluated twice by two different observers. The arithmetical mean of the two punctuations was taken as the final score.

Immunohistochemical staining of iNOS in paraffin sections (4 μm) was performed using an avidin-biotin peroxidase technique as described by Shu el al. [27], with some modifications. The primary antibody anti-iNOS (1:500) was obtained from Oxford Biomedical Research, INC, NS 01. The procedure for negative controls was performed by omission of antigen retrieval part of the protocol. The positive staining was estimated blindly in the entire preparation by using a numerical scored from 1 to 8 points attending to the staining area and the intensity of the color. The arithmetical mean of the two evaluations was taken as the final score.

### Analytical methods

#### Sample processing

Hepatic samples were homogenized in a Potter homogenizer in 7 volumes of cold buffer (0.1 M Tris-HCl, 0.25 M sucrose, pH= 7.4) containing 5 mM 2-mercaptoethanol, 0.5 μg/mL Leupeptin, 0.7 μg/mL pepstatin A and 100 μg/mL PMFS. Fibrous parts and unbroken cells debris were eliminated by centrifugion at 500 g for 5 min. Supernatans were used as the whole homogenate.

#### Analytical determinations on hepatic homogenates

MPO was measured by ELISA, using a commercial kit from BIOXYTECH^® ^(OXIS Int. Portland, OR, USA). Transferrin and ferritin were determined by immunoturbidimetry, using a Hitachi 710 autoanalyzer (Roche Diagnostic, Basilea, Switzerland) and kits for clinical human, from the same laboratory. MDA was assessed after heating samples at 45°C for 60 minutes in acid medium. It was quantitated by a colorimetric assay using LPO-586 (Bioxytech; OXIS International Inc., Portland, OR, USA), which after reacting with MDA, generating a stable chromophore that can be measured at 586 nm (Hitachi U2000 Spectro; Roche). Total proteins were assessed by Bradford's method [28].

#### Determinations of iron and copper by Atomic Absorption Spectrophotometry

Representative samples (approximately 1 g. of each rat liver) were collected, weighed and later dried in stove (70°C) to constant weight. Iron and copper concentrations were determined by flame atomic absorption spectrophotometry (Perkin Elmer 460, Uberlingen, Germany) [25].

### Statistical Analysis

Data were expressed as mean ± SEM. To analyse the homogeneity among groups, Kruskall-Wallis test was used, followed by multiple post-hoc comparisons using Mann-Whitney U tests with Bonferroni adjustment. Any *P *value < 0.05 was considered to be statistically significant. Calculations were performed with SPSS program version 6.0 (SPSS Inc., Chicago, IL).

## Results

Liver cirrhosis was histologically proven and ascites was observed in all rats treated with CCl_4_.

Table 1 shows the values of parameters involved in oxidative damage in hepatic homogenates. Compared with healthy controls, untreated cirrhotic rats (CI group) showed increased hepatic levels of the following variables: Fe (p < 0.01); transferrin and ferritin (p < 0.01); Cu (p < 0.001); MPO and iNOS expression (p < 0.001). However, cirrhotic animals treated with IGF-I (CI+IGF group) showed significant reductions in hepatic Fe and Cu contents, ferritin, transferrin and MPO levels and iNOS expression (p < 0.05 for all the parameters).

**Figure 1:**
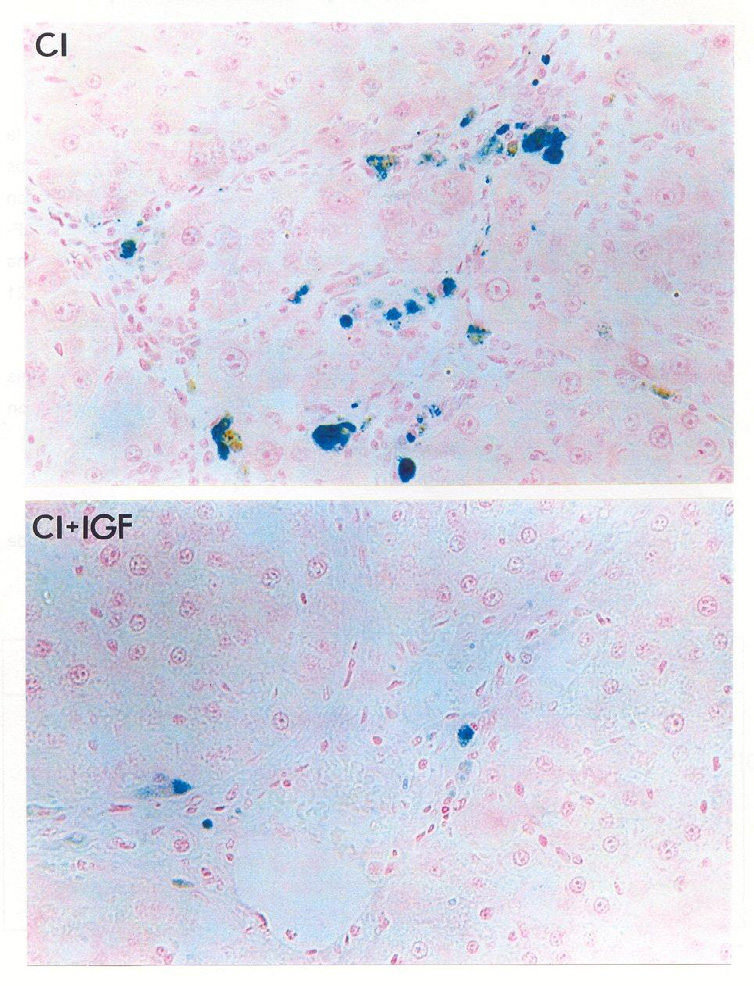
Perl's Prussian Blue staining for ferric iron (original magnification ×150) in the liver of an untreated cirrhotic rat (CI group) and a cirrhotic animal treated with IGF-I. The CI preparation was scored as 3 points (see Methods) and the section from CI+IGF group was scored with 1 point. No staining was found in control group (CO).

As shown in Figure 1, untreated cirrhotic rats (CI) have significantly greater scores of iron (ferric iron) in the liver using Perls' Prussian blue staining as compared with controls (CO = 0.68 ± 0.11) and cirrhotic rats treated with IGF-I (CI = 5.50 ± 0.22; CI+IGF = 1.70 ± 0.40; AU, p < 0.01). As mentioned before, hepatic levels of iron, assessed by atomic absorption spectrophotometry, were also significantly higher in CI group compared to controls and CI+IGF group (see Table 1). On the other hand, hepatic levels of copper were also increased in untreated cirrhotic rats and returned to normal in CI+IGF group.

**Figure 2:**
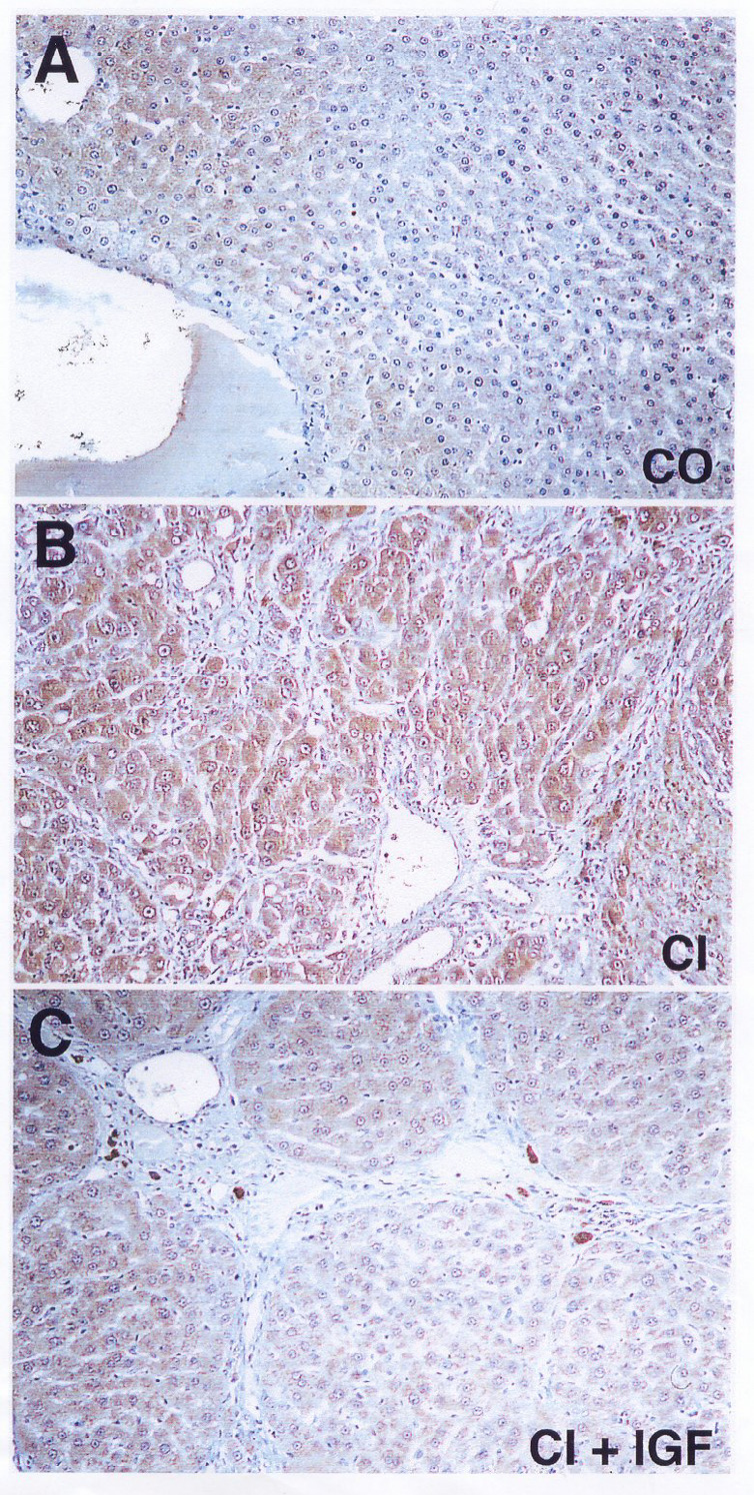
Immunostaining for iNOS in liver from: A, healthy control group (CO); B, untreated cirrhotic group (CI); C, cirrhotic animals treated with IGF-I for three weeks. An increased iNOS immunoreactivity was observed in hepatocytes from CI group, compared to controls and CI+IGF groups. These two pictures (B and C) correspond to two animals from each cirrhotic group that presented the most severe cirrhosis. Although in this section (C, CI+IGF) from a series with decompensated cirrhosis can be observed thick collagen septa, it is also clear the hepatoprotective effect of the IGF-I-therapy versus untreated cirrhotic group (B).

Figure 2 shows the immunohistochemical expression of iNOS that was increased in CI group compared both to control and CI+IGF groups.

**Figure 3:**
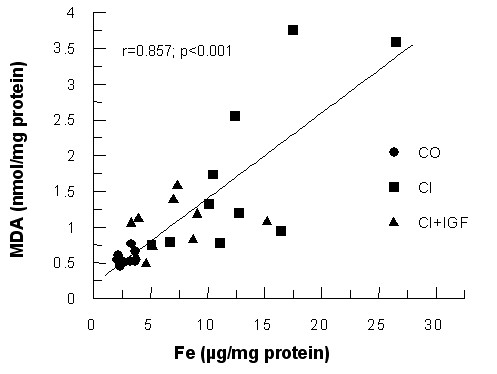
Correlation between hepatic iron content and hepatic MDA levels, a marker of lipid peroxidation (Sperman r = 0.857, p < 0.001, two tails).

In order to find a relationship between the studied parameters and oxidative liver damage, MDA levels, an index of lipid peroxidation, were evaluated [29]. Hepatic levels of MDA (nmol/mg protein) were increased in untreated cirrhotic rats compared with control group (CI = 1.741 ± 366; CO = 0.565 ± 0.030; p < 0.05) as it was previously reported in similar protocols [14,16]. This marker of lipid peroxidation was again reduced in CI+IGF (0.99 ± 0.11 nmol/mg protein, p = ns vs controls). A significant direct correlation was found between hepatic iron and hepatic MDA levels (see Figure 3, r = 0.857 p < 0.001). In addition, MPO correlated with hepatic levels of iron (r = 0.719, p < 0.001), iron content with hepatic ferritin (r = 0.656, p < 0.001) and hepatic levels of Cu with MDA (0.649 p < 0.01).

## Discussion

These results show that the treatment with low doses of IGF-I induces a reduction of all studied parameters involved in oxidative damage mechanisms in this model of cirrhosis. These findings support the hepatoprotective and antifibrogenic effects previously reported [14,16]. This study also provides evidence for the involvement of oxidative stress in the cell injury occurring in CCl_4_-induced cirrhosis associated with iron and copper overload and an increase of myeloperoxidase and iNOS expression.

It is well known that iron and copper promote oxidant forces [17,18,21,30]. Oxidant stress is considered present when there is either an overproduction of free radicals or a significant diminution in antioxidant defenses, the result of either being excessive levels of free radicals [29,31]. In both iron and copper storage disorders, generation of free radicals and depletion of antioxidants may be critical factors determining the intensity of liver injury [18,19,30,31]. In a previous work we showed that antioxidant enzymes (superoxide dismutase, SOD, Glutathione peroxidase, GSHPx, and catalase) were reduced in the liver of cirrhotic animals and improved by low doses of IGF-I administration [14]. Of interest, in the present study we demonstrate that hepatic levels of iron and copper metals (both involved in oxidative damage), increased in untreated cirrhotic rats, reverted to normal levels after IGF-I treatment.

Free iron (or low molecular iron or chelatable iron pool) facilitates the decomposition of lipid hydroperoxides resulting in lipid peroxidation and induces the generation of OH radicals and also accelerates the nonenzymatic oxidation of glutathione to form O_2_*- radicals [18,19,30,32]. The direct and significant correlation between lipid peroxidation and hepatic iron content presented here provides new evidence of the relationship between these parameters.

Most of the body's iron is tightly bound to transferrin, entering cells via receptor-mediated endocytosis. Transferrin avidly binds 2 moles of Fe^3+ ^per mole of protein [32]. Normally the average of transferrin iron saturation is about one third of the full capacity, thereby ensuring that there is virtually no free iron circulating in the extracellular fluids. At pH 7.4, the iron-transferrin complex does not participate in the Fenton reaction. Under more acidic conditions, the complex breaks down with release of iron. This is of important physiological relevance, since the iron-transferrin complex, within endocytotic vesicles, is subjected to an acidic environment (pH 5–6). Intracellular iron released from transferrin is rapidly incorporated into ferritin, minimizing its inherent toxicity [17,30,31]. Iron can be released from the ferritin within the cell by a number of factors that occur in inflammation: acidic pH, proteolysis, myeloperoxidase, NO, O_2_*-, etc. [33]. Enhanced degradative proteolysis, which also occurs in oxidative stress, may lead to proteolytic modification of ferritin, causing an increase in cellular iron. Although in this study free iron could not be quantified, all of the factors certainly involved in inducing an increase of free iron pool appeared elevated in untreated cirrhotic rats (MPO, iNOS, Cu,...) and returned to normal levels after IGF-I-treatment.

In the present study, we have found that hepatic transferrin and ferritin levels increased in cirrhotic rats with a parallel rise in iron deposition, whereas in cirrhotic rats treated with IGF-I all the above-mentioned parameters appeared diminished (see Table 1 and Figure 1).

High serum ferritin levels and hepatic iron storage have also been reported in hepatitis B virus and hepatitis C virus-related chronic hepatitis and alcoholic liver disease [26,34,35]. It has also been shown that iron induces ferritin biosynthesis [21,22,35-39]. A result here presented shows a direct correlation between hepatic iron and ferritin levels which is consistent with the over-mentioned Authors statements.

In liver cirrhosis the increase in iron content is not a real iron overload as in hemochromatosis, because iron is stored mainly inside the macrophages [40]. In agreement with this data, the present work shows that the iron scores detected in this experimental model of cirrhosis were found in Kupffer cells, as it is shown in Fig. 1.

Transferrin is mainly produced by the liver when hepatic regeneration takes place, as occurs in cirrhosis [37,41,42]. Thus, the reported increase of transferrin in untreated cirrhotic animals could be due to regeneration. However, cellular proliferation does not explain our findings, because in a parallel study in this series we showed that cellular proliferation (assessed by PCNA expression) was higher in IGF-treated cirrhotic animals [15] than those which showed lower hepatic levels of transferrin. Therefore, the hepatoprotective effect of IGF-I in cirrhotic animals could be mediated partly by enhancing the endogenous regenerative response, aimed al the restoration of functional liver mass [14]. In the present work, the described increase of transferrin in untreated cirrhotic animals seems to be a defensive response to the enhanced iron content [17,18,21,22,32].

On the other hand, the mechanisms responsible for the effects of IGF-I described in this article are not fully understood. The beneficial effects of IGF-I could be a result of many properties of this hormone that require further investigation. The well known erythropoietic activity of IGF-I [43,44] could even contribute to an extrahepatic utilization of iron, decreasing its storage in the liver.

Hepatic copper overload leads to progressive liver injury and eventually cirrhosis in Wilson disease and Indian childhood cirrhosis [45]. Copper is absorbed into the intestine and transported by albumin to the liver. Any excess in copper levels is excreted into the bile mainly through a lysosome-to-bile pathway. Hepatic copper accumulation results from a reduction in the bile excretion of copper, as occurs in patients with Wilson disease, biliary obstruction, or other types of cholestasis [45]. Cirrhotic animals included in this protocol showed severe cholestasis after receiving CCl_4 _for 30 weeks. As previously reported [14] IGF-I-treatment induced a reduction in cholestasis parameters (serum levels of bilirubin, alkaline phosphatase and cholesterol). This may account for explanation of the decreased copper hepatic content revealed in the present work.

After hepatic injury, several kinds of cells (endothelial cells, Kuppfer cells, and circulating platelets, neutrophils and monocytes) are activated in the subsequent inflammatory response [23]. Free radicals produced mainly by macrophages cause local tissue damage in inflammatory conditions [23,31]. Neutrophil and monocyte activation is a critical step in both the host defense system against microorganisms and the inflammatory response. When neutrophils are activated, they begin to produce superoxide radicals (O_2_*-) and secrete myeloperoxidase (MPO) [23]. The majority of the O_2_*- formed during this respiratory burst is converted to the bactericidal oxidant hypochlorous acid (HOCl) via a series of reactions catalyzed by superoxide dismutase and MPO [23,29]. Numerous MPO-expressing cells have been detected in fibrous septa of human cirrhotic livers [46]. MPO has been identified as a component of human Kupffer cells [46]. The same authors suggest that the oxidative damage resulting from the action of MPO may contribute to acute liver injury and hepatic fibrogenesis [46]. In our study, the increase of MPO in cirrhotic animals and its decrease in those treated with IGF-I suggests an anti-inflammatory effect of this hormone.

Another result which deserves particular mention is that iNOS expression was significantly lower in cirrhotic rats treated with IGF-I compared to untreated cirrhotic animals. This finding is in accordance with those reported by other groups [47-53]. However the versatility of this molecule, small changes in the experimental conditions or the studied cell line can show results that seem to be an apparently contradiction [54-58]. For example, in our experience, we did not find a similar response in early stage of cirrhosis animals (data not shown). Probably, in early stages of cirrhosis NO induces an improvement in parenchyma irrigation by vasodilatation, but in advanced liver cirrhosis, where exist thick collagen septa, the increase of NO results to lead enhancing oxidative damage by N-derived radicals.

## Conclusion

In conclusion, these results show that the hepatoprotective and antifibrogenic effect of IGF-I in rats with liver cirrhosis is associated with a significant reduction of the hepatic levels of several parameters such as Fe, Cu, MPO, iNOS, ferritin and transferring, all of them involved in oxidative damage. In this work, iron and copper overload have been demonstrated in the liver from rats with CCl_4_-induced cirrhosis. The hepatic levels of both metals diminished in cirrhotic animals treated with IGF-I. MPO content, iNOS immunohistological expression and hepatic ferritin and transferrin levels were increased in untreated animals and returned to normal in cirrhotic animals treated with IGF-I.

The IGF-I effects described in the present study suggest that a therapeutical approach targeted at lowering oxidative stress marker levels could be effective in the chronic liver disease.

## Abbreviations

IGF-I, insulin-like growth factor-I; Fe, iron; Cu, cooper; MDA, malondialdehyde; CO, control healthy group; CI, untreated cirrhotic rats; CI + IGF, IGF-treated cirrhotic rats; O_2_*-, superoxide radicals; MPO, myeloperoxidase; iNOS, inducible nitric oxide synthase; AU, arbitrary units.

## Competing interests

The author(s) declare that they have no competing interests.

## Authors' contributions

**MG**: Analytical studies, hypothesis and paper elaboration.

**ICC**: Experimental design and treatment (induction of liver cirrhosis and IGF-I administration), hypothesis, histopathological study and scores.

**MDS**: Analytical studies and in vivo assay.

**IN**: Atomic absorption spectrometry assay.

**JEP**: In vivo assay.

**AC**: Hypothesis and experimental design and revision.

**ADC**: Experimental treatment and documentation.

**EC**: Histopathological study and measurements.

**SGB**: Revision.

## Pre-publication history

The pre-publication history for this paper can be accessed here:



## Figures and Tables

**Table 1:** Hepatic levels of some parameters involved in oxidative damage in the three experimental groups.

	**Control group (CO, n = 10)**	**Untreated cirrhotic rats (CI, n = 10)**	**Cirrhotic rats treated with IGF-I (CI+IGF, n = 10)**
Fe (μg/mg protein)	2.74 ± 0.19	12.87 ± 1.90**	6.80 ± 1.10^&^
Cu (μg/mg protein)	160 ± 5	1626 ± 678***	500 ± 258^&^
Ferritin (ng/mg prot)	32.60 ± 3.80	97.60 ± 12**	67.70 ± 11.30^&^
Transferrin (μg/mg protein)	8.46 ± 1.05	10.96 ± 0.98**	8.36 ± 0.36^&^
MPO(ng/mg protein)	1.02 ± 0.02	1.25 ± 0.04**	1.06 ± 0.07^&^
iNOS (AU)	1.20 ± 0.64	5.53 ± 0.54***	2.88 ± 0.68^&^

^&^p < 0.05 between CI and CI+IGF groups; **p < 0.01 and ***p < 0.001 CI vs CO groups; AU = arbitrary units
